# Shrinkage Stress, Polymerization Kinetics, and Hardness of Light and Self-Cured Bulk-Fill Resin-Based Composites

**DOI:** 10.3390/ma19122623

**Published:** 2026-06-18

**Authors:** Raphaël Decroos, Cristiane Maucoski, Brett D. MacNeil, Darien DeWolf, Daniel Labrie, Richard B. Price

**Affiliations:** 1 Department of Physics and Atmospheric Science, Dalhousie University, Halifax, NS B3H 4R2, Canada; raphael.decroos@2025.icam.fr (R.D.); daniel.labrie@dal.ca (D.L.); 2 Department of Dental Clinical Sciences, Dalhousie University, 5981 University Avenue, Halifax, NS B3H 4R2, Canada; cmaucoski@dal.ca; 3 Department of Mathematics and Statistics, St. Francis Xavier University, 4130 University Avenue, Antigonish, NS B2G 2W5, Canada; ddewolf@stfx.ca

**Keywords:** bulk-fill composite resins, polymerization, shrinkage stress, degree of conversion, Vickers hardness

## Abstract

**Highlights:**

- Shrinkage stress differed among the six resin-based composites.
- Self-cured resin-based composites developed stress more slowly.
- Degree of conversion and hardness did not correlate with shrinkage stress.

**Abstract:**

The polymerization shrinkage stress (SS), degree of conversion (DC), and Vickers hardness (HV) are properties that can affect the performance of resin-based composites (RBCs). This study tested four bulk-fill RBCs used in self-cured mode: Bulk EZ Plus (Zest Dental Solutions), Cention Forte (Ivoclar), Fill-Up! (Coltene), and Stela (SDI Limited), and two light-cured bulk-fill RBCs: Filtek One (Solventum) and SDR flow+ (Dentsply). The test specimens were 6 mm in diameter and 2 mm thick. Axial SS was measured in real time for 4000 s in the self-cured materials and for 1400 s after 10 s of light curing in the light-cured materials (n = 12 for self-cured RBCs; n = 11 for light-cured RBCs). To confirm that the RBCs were adequately polymerized, the DC was assessed using real-time ATR-FTIR spectroscopy, and the HV was measured on the top and bottom surfaces using a 300-gf load for 8 s (n = 5) after 24 h. The SS, DC, and HV differed significantly among the RBCs (*p* < 0.001). At 1400 s, Cention Forte developed the lowest stress (1.44 MPa), whereas Bulk EZ Plus and Fill-Up! produced the highest stress (3.77 MPa). The self-cured materials continued to develop measurable stress between 1400 s and 4000 s, while the light-cured RBCs had stabilized at 1400 s. Bulk EZ Plus and Stela produced the highest DC values, and Stela had the highest HV.

## 1. Introduction

Light-cured resin-based composites (RBCs) have become the material of choice for direct dental restorations because of their excellent aesthetic properties and ability to replicate the optical characteristics of natural teeth [1,2,3]. However, the effects of polymerization shrinkage of RBCs are a major challenge [1,4,5,6,7,8,9]. The volume reduction that occurs when monomers convert into polymer chains creates strain within the restoration and stress at the tooth-restoration interface [1,7]. For example, a 1% linear shrinkage across a 10 mm wide restoration represents a 100 μm dimensional change in the RBC restoration. This may contribute to bond failure [6,10], microleakage, debonding, secondary caries, enamel cracking, post-operative hypersensitivity, marginal defects, compromised mechanical properties, increased wear, reduced longevity, and premature failure [5,6,10,11]. Thus, studying the shrinkage and shrinkage stress (SS) produced by different RBCs is clinically relevant [5,6,7,8,11].

Axial shrinkage and shrinkage stress are distinct, yet interconnected aspects of polymerization dynamics [12] that are both RBC- and cavity-geometry-dependent [1,13,14]. The monomer, filler content, DC, reaction kinetics, and thickness all influence the development of shrinkage stress [6,9,15,16,17,18,19,20,21]. Rapid polymerization kinetics further complicate the management of shrinkage stress in light-cured RBCs [1,6,7,15]. When a high irradiance is delivered, polymerization occurs very rapidly, leaving little time for any flow or relaxation before the network becomes rigid [5,16,22,23,24]. The amount of light reaching the bottom of the restoration is also a challenge in deep cavity preparations [19,20,25,26,27] because, in accordance with Beer’s Law, the intensity of transmitted light decreases exponentially as RBC thickness increases. Even at high irradiance, this exponential decrease in light intensity means that inadequate polymerization of conventional RBCs may occur when the thickness of the RBC exceeds 1.5 to 2 mm [28]. The effects of this light attenuation are also greater in the violet (<410 nm) than in the blue wavelength region (~460 nm) [1]. This can produce non-uniform polymerization and internal stress gradients [23] because RBCs closer to the light source photocure sooner than the RBCs at the bottom of the restoration [19,20,25,26,27].

Axial shrinkage quantifies the dimensional contraction during polymerization and can be measured using dilatometry, bonded-disk, or optical methods [5,29]. Shrinkage stress quantifies the force generated when contraction is constrained by bonding to cavity walls and is influenced by the C-factor, the modulus of the RBC that develops during polymerization, and the modulus of the surrounding tooth structure [1,8]. Although different force transducer systems are widely used [4], stress has also been measured using a load cell to determine the extent of cusp or model tooth deflection, or by observing cracks in the enamel [5].

The amount of shrinkage stress depends strongly on the viscoelastic state of the RBC as it polymerizes. RBCs that develop a high modulus during early polymerization will generate more stress per unit shrinkage than materials whose modulus increases more gradually [8,30,31]. Therefore, the rate of modulus development, not only the final modulus, is a critical determinant of stress formation, and RBCs that remain viscous or viscoelastic for longer can undergo more flow and stress relaxation. This can partially compensate for the polymerization contraction that occurs before the modulus reaches a level that limits molecular mobility [31,32,33]. Rapid photopolymerization at high irradiance can accelerate conversion and network formation, thereby reducing the time available for stress-relieving flow while the dimensional change and modulus development occur concurrently [1,16,22,34]. Thus, RBCs with similar shrinkage strain values can generate very different stress levels.

Self-curing and dual-curing RBCs can offer a fundamentally different polymerization timescale from light-cured RBCs. Dynamic mechanical analysis has shown that, during self-curing, resin-based composites often exhibit a gradual increase in storage modulus (E′) relative to the loss modulus (E″), indicating a progressive transition from predominantly viscous to more elastic behavior [35]. Because the modulus develops more slowly during self-curing, the self-cure RBC may have more time for viscous flow and stress relaxation before gelation/vitrification. This can partially compensate for dimensional changes [12,22,36,37] and reduce the final shrinkage force compared with more rapid polymerization protocols [38,39]. Thus, in high C-factor cavities, where geometry restricts stress relief by flow, the extended pre-gel phase of self-cured RBCs may help preserve marginal integrity, reduce interfacial debonding, and improve clinical success. Consequently, some contemporary self-curing and dual-cure bulk-fill RBCs have been reported to be promising alternatives to light-cured restorative materials [40,41].

Cention Forte (Ivoclar) and Stela (SDI Limited) do not require a curing light and are marketed as self-curing RBCs that can be used as alternatives to amalgam. Stela has a distinct polymerization profile in which polymerization starts at the bonding interface inward [42], and two favorable clinical trials have been reported for Stela [40,41]. This RBC is available in both automix (dual-paste) and capsule formulations that differ in filler content and viscosity. Cention Forte is an alkasite-based restorative material [43] that primarily relies on a self-curing polymerization strategy, although optional light activation may also be used. Cention Forte is supplied in capsule form and must be triturated in a mechanical mixer. Fill-Up! (Coltene) and Bulk EZ Plus (Zest Dental Solutions) are dual-cured bulk-fill RBCs supplied as automix systems. The instructions for use state that Fill-Up! and Bulk EZ Plus can self-cure without light activation, although light curing may be used to accelerate the curing process. Since these RBCs differ substantially in the composition of their resin matrix, filler loading, and viscosity, all of which might affect polymerization kinetics and properties [12], the relationship between polymerization kinetics and stress development requires investigation. Therefore, this study investigated the real-time stress development of six RBCs. To confirm that the RBCs were adequately polymerized, the degree of conversion and Vickers hardness were also measured. The null hypotheses were:

(1) There will be no difference in the shrinkage stress between self-cured and light-cured RBCs at 1400 s.
(2) There will be no difference in the degree of conversion between self-cured and light-cured RBCs.
(3) There will be no difference in the hardness among the six RBCs after 24 h of storage, and no difference between the self-cured and light-cured materials.

## 2. Materials and Methods

### 2.1. Materials

The information provided by the manufacturers of four self-cured and two light-cured bulk-fill RBCs tested in this study is reported in Table 1. The four RBCs used in self-cured mode were Stela Capsule (SDI, Bayswater, Australia), Cention Forte (Ivoclar, Schaan, Liechtenstein), Bulk EZ Plus (Zest Dental Solutions, Carlsbad, CA, USA), and Fill-Up! (Coltene, Altstätten, Switzerland). Although Fill-Up! and Bulk EZ Plus are marketed as dual-cure composites, their respective Instructions for Use (IFUs) indicate that the light-curing step is optional, but it does accelerate surface hardening, reduce the oxygen-inhibited layer, and allows finishing sooner. The two light-cured RBCs used as controls were SDR flow+ (Dentsply Sirona, Milford, DE, USA) and Filtek One (Solventum, St. Paul, MN, USA).

### 2.2. Axial Shrinkage Stress (SS)

**Figure 1:**
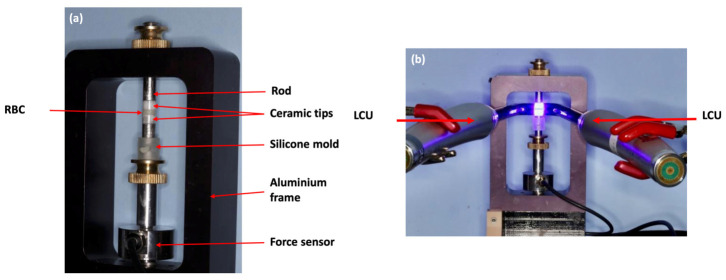
(**a**) Components of the Ultradent shrinkage stress testing device. (**b**) Two LED-based light-curing units (LCUs) were used on each side of the 6-mm diameter sample to photocure the light-cured RBCs.

The axial SS was measured using the Ultradent shrinkage stress tester (Ultradent Products Inc., South Jordan, UT, USA), which quantified polymerization-induced stress along a single axis. As shown in Figure 1a, the device consisted of two stainless steel rods that had 6 mm diameter zirconia tips and corresponding nuts to secure the top rod to the aluminum frame and the bottom rod to the force transducer. This enabled the shrinkage-induced stress to be recorded in real time (every 0.06 s). The gap between the two zirconia surfaces was set to 2 mm using a digital gauge (Mitutoyo 543-456B, Mitutoyo Canada Inc., Mississauga, ON, Canada). Figure 1b shows the two LED-based LCUs (Elipar DeepCure-S, Solventum, St. Paul, MN, USA) positioned on either side for simultaneous light curing of Filtek One and SDR flow+. The LCU tips were parallel to each other, centered on the specimen, and positioned less than 1 mm from the surface of the RBC.

Before insertion of the RBC, the zirconia disc surfaces were air-abraded with 50 µm alumina powder. A light-cured dental adhesive (Scotchbond Universal Plus; Solventum, St. Paul, MN, USA) was applied to the zirconia and photocured according to its instructions for use. A cylindrical silicone sleeve with an injection port was positioned over the zirconia tips, and the RBCs were injected into the space between them. This sleeve facilitated specimen placement and ensured complete filling of the space. After insertion, the sleeve was slid along one rod to expose the RBC sample, which was then shaped into a cylinder between the two ceramic tips using a dental instrument. For the self-cured RBCs, the stress measurements began immediately after the sleeve was removed. For the light-cured RBCs, stress measurements began shortly before the LCUs were turned on. The axial SS was monitored for 4000 s after the application of self-cured RBCs from their mixing tips, whereas for light-cured RBCs, the samples were measured for 1400 s. The rationale for measuring light-cured RBCs only up to 1400 s was that there was a rapid increase in SS upon exposure to light that reached a plateau substantially earlier than in the self-cured RBCs. The SS values at fixed time points, maximum stress rate, the time of maximum stress rate, and the stress at the time of maximum stress rate were extracted for each repeat and summarized for each RBC as the mean and standard error (SE). These values were based on 12 repeated measurements per RBC; for SDR flow+ and Filtek One, stress values at 1400 s were based on 11 repeats, and stress-rate-based metrics were not applicable due to their rapid photocuring behavior.

### 2.3. Light Output

The light output from the two LED-based Elipar DeepCure-S LCUs was measured using a laboratory-grade fiberoptic spectrometer (Ocean SR, Ocean Optics, Orlando, FL, USA) attached to a 15 cm diameter integrating sphere (Labsphere, North Sutton, NH, USA) that had a 16 mm aperture into the sphere. As described previously [44], the LCU tip was positioned at the entrance of the integrating sphere, which had been calibrated with an internal calibration source (ICS-600, Labsphere), and the emitted light was recorded with OceanView version 2.0 software (Ocean Optics, Orlando, FL, USA) from 350 to 1100 nm. The active tip diameter and optical emission area of the light tip were measured. The radiant power (mW) was divided by the emission area (cm^2^) to calculate radiant exitance (mW/cm^2^). Three recordings were made for each LCU (n = 3).

### 2.4. Degree of Conversion (DC)

The mid-infrared absorption spectra from the six RBCs were collected at room temperature (23 °C) in real time using a Vertex 70 Fourier Transform Infrared (FT-IR) spectrometer (Bruker, Billerica, MA, USA) with a temperature-controlled Attenuated Total Reflectance (ATR) diamond prism attachment (Specac, Orpington, Kent, UK). The uncured RBC specimens were placed into 6 mm diameter by 2 mm thick disks directly on the ATR diamond prism and covered with a 50 µm thick polyester sheet. For SDR flow+ and Filtek One, the tip of the LCU light guide was positioned directly above the RBC at a 0 mm distance, and the RBCs were photocured for 10 s.

Opus version 8.7 (Bruker) was used to collect the spectra using the double-sided, forward-backward data-collection mode, with a spectral resolution of 8 cm^−1^ and a spectral range of 1250–1800 cm^−1^, resulting in approximately 13 DC data points per second. For three RBCs (Bulk EZ Plus, Fill-Up! and SDR flow+), the DC was calculated by comparing the spectral peak absorption height at time t, H(t), for the aliphatic methacrylate (C = C) peak (1637 cm^−1^) with that of an aromatic absorption peak (1609 cm^−1^) for Bulk EZ Plus and Fill-Up! and (1602 cm^−1^) for SDR flow+, with both peaks being normalized with respect to their heights, H(t = 0), in their reference spectra collected before the start of polymerization (defined as t = 0 s), and then taking the ratio of these two values:

(1) $$DC\left(t\right)=100\%\left(1-\left(\frac{{H(t)}_{1637}/{H(t=0)}_{1637}}{{H(t)}_{1609\;or\;1602}/{H(t=0)}_{1609\;or\;1602}}\right)\right)$$

Stela, Cention Forte, and Filtek One did not have identifiable aromatic peaks. Therefore, the DC was calculated by comparing changes in the monomer absorbance spectrum alone [45,46] by using the ratio of the height of the aliphatic C=C peak (1637 cm^−1^) at time *t*, *H*(*t*), to its height in the reference spectrum, *H*(*t* = 0):

(2) $$DC\left(t\right)=100\%\left(1-\left(\frac{{H\left(t\right)}_{1637}}{{H\left(t=0\right)}_{1637}}\right)\right)$$

For the self-curing RBCs, the start time of the reaction was estimated by using the point of inflection (the maximum of the second derivative) of the conversion curve and extrapolating the tangent to the time axis at zero DC. For the light-curing RBCs, the measurement began when the LCU was switched on. The DC was recorded for 1800 s (30 min) in real time for all six RBCs, and five repetitions were made in each group (n = 5). Due to time scaling, for the self-cured RBCs, the DC values are reported at 1600 s after the onset of polymerization. The DC(*t*) curves were used to compute DC at 1600 s, the maximum DC rate, the time of maximum DC rate, ${\left.dDC/dt\right|}_{t_{0}}$ *t*_0_, and the DC at the time of maximum rate, DC(*t*_0_).

### 2.5. Autocatalytic Kinetic Model

The DC rate data were fitted to the autocatalytic model [47]. This kinetic equation relates the polymerization rate to the DC using the differential equation:

(3) $$\frac{d}{dt}\left(DC\left(t\right)\right)=k{\left(DC\left(t\right)\right)}^{m}\;{\left({DC}_{max}-DC\left(t\right)\right)}^{n}$$

where *k*, *m*, and *n* are fitting parameters. The parameter DC_max_ was set to the value reached at the end of data collection. Before fitting, the rate data were smoothed using a linear Savitzky–Golay filter with an averaging window of 300 ms for light-cured RBCs and 2 s for self-cured RBCs [47,48]. Each of the n = 5 measurements for each RBC was independently fitted to the model. The data were also interpolated and averaged to produce an average DC-rate curve.

### 2.6. Vickers Hardness (HV)

After the DC measurements, the RBC specimens were stored in the dark for 24 h at 37 °C in air, at 55% ambient humidity. The Vickers hardness (HV) values were measured with a microhardness tester (HM-200, Mitutoyo Canada Inc., Mississauga, ON, Canada) using a load of 300 gf for 8 s [49]. Five indentations were made in the middle of the top and bottom surfaces of the specimens with a 1 mm separation between each indent, and an average HV value was calculated for each surface.

### 2.7. Statistical Analysis

The study factor was the six RBCs: Bulk EZ Plus, Cention Forte, Fill-Up!, SDR flow+, Stela Capsule, and Filtek One. For the hardness analysis, an additional factor was the surface, with two levels (top and bottom). The response variables were SS, maximum stress rate, time of maximum stress rate, stress at maximum stress rate, DC, maximum DC rate, time of maximum DC rate, DC at maximum rate, and HV.

Inferential statistics were conducted to determine whether statistically significant differences existed among the RBCs for each outcome measure. For every outcome, assumptions of normality were evaluated using the Shapiro–Wilk test applied to each group’s data and to the one-way ANOVA residuals; homogeneity of variances was assessed using Levene’s test (median-centered). When both assumptions were satisfied, a one-way ANOVA was used for omnibus testing, followed by Tukey’s honest significant difference (HSD) post hoc test for pairwise comparisons. When normality was satisfied, but variances were unequal (Levene’s *p* < 0.05), Welch’s ANOVA was used with Games–Howell post hoc tests. When normality was violated in one or more groups, a Kruskal–Wallis test was used, with Dunn’s test for pairwise comparisons and Holm correction for multiple comparisons. In cases where per-group normality held but the ANOVA residuals exhibited non-normality alongside significantly unequal variances, the residual non-normality was attributed to heteroscedasticity, and Welch’s ANOVA was applied (e.g., maximum stress rate). Effect size was estimated using η^2^ (when using non-parametric tests, the rank-based η^2^ was used). The family-wise error rate was controlled within each outcome family; no global correction across the fifteen outcome families was applied. Compact letter displays (CLD) were used to summarize pairwise group membership. For hardness, the top versus bottom surface within each RBC was assessed using paired *t*-tests (or Wilcoxon signed-rank tests where differences were non-normal), with Bonferroni correction applied across the six within-RBC comparisons. Similarly, for shrinkage stress, the difference between measurements at 1400 s and 4000 s was assessed within each self-cured RBC using paired *t*-tests (or Wilcoxon signed-rank tests where differences were non-normal), with Bonferroni correction applied across the four within-RBC comparisons. Statistical significance was set at α = 0.05.

A priori power analysis was conducted using the package pwr version 1.3-0 to determine the minimum detectable effect size at 80% power for each design configuration. Minimum detectable effect sizes were also converted to real measurement units using pooled within-group standard deviations. Post hoc observed power was computed for completeness [50], and the SS, DC, and HV omnibus tests had observed powers of 1.000. All analyses were conducted in R (version 4.5.1) [50] using the packages readxl version 1.4.5, dplyr version 1.1.4, tidyr version 1.3.1, car version 3.1-4, rstatix version 0.7.2, effectsize version 1.0.1, multcompView version 0.1-10, pwr 1.3-0, and gt 1.0.0.

## 3. Results

### 3.1. Light Output 

**Figure 2:**
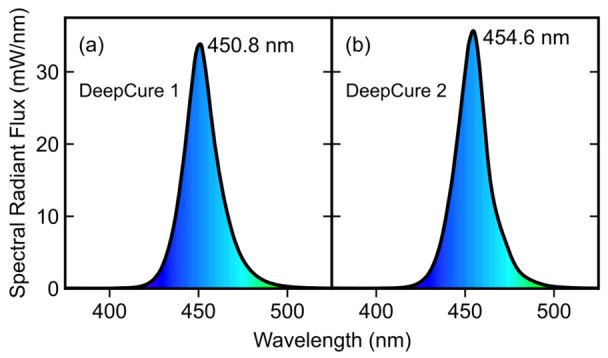
Spectral radiant flux (mW/nm) from the two Elipar DeepCure-S LCUs.

Figure 2 illustrates the spectral radiant flux from the two Elipar DeepCure-S LCUs. Each was a single-peak LED-based LCU with one wavelength peak in the blue region (λ = 451 nm for LCU #1 and λ = 455 nm for LCU #2). The emitted power was 813 ± 1.2 mW from LCU #1 and 820 ± 0.5 mW from LCU #2.

Based on the active tip diameter (9.1 mm), the radiant exitance was 1.249 W/cm^2^ for LCU #1 and 1.261 W/cm^2^ for LCU #2. Thus, a radiant exposure of 12.5 J/cm^2^ was delivered from one side, and 12.6 J/cm^2^ from the other side of the RBC after 10 s. These values were well within the manufacturer’s specifications.

### 3.2. Axial Shrinkage Stress (SS)

Table 2 reports the shrinkage stress parameters and statistical tests. The SS varied significantly among the six RBCs for all outcomes (*p* < 0.001). At 1400 s, Cention Forte produced the lowest stress (1.44 MPa), whereas Bulk EZ Plus and Fill-Up! generated the highest stress, which was approximately 2.6 times greater (3.77 MPa). Among the self-cured materials, the increase in stress from 1400 to 4000 s was the greatest for Cention Forte and the lowest for Bulk EZ Plus and Fill-Up! The maximum stress rate also differed markedly among materials (*p* < 0.001): Bulk EZ Plus and Fill-Up! produced the highest rates (0.0130 and 0.0113 MPa/s, respectively), followed by Stela, whereas Cention Forte produced the lowest rate (0.0016 MPa/s). The time to maximum stress rate was shortest for Bulk EZ Plus and Stela (132.09 and 147.41 s, respectively) and longest for Cention Forte (432.36 s). The stress at maximum rate was highest for Fill-Up!, intermediate for Stela and Bulk EZ Plus, and lowest for Cention Forte.

**Figure 3:**
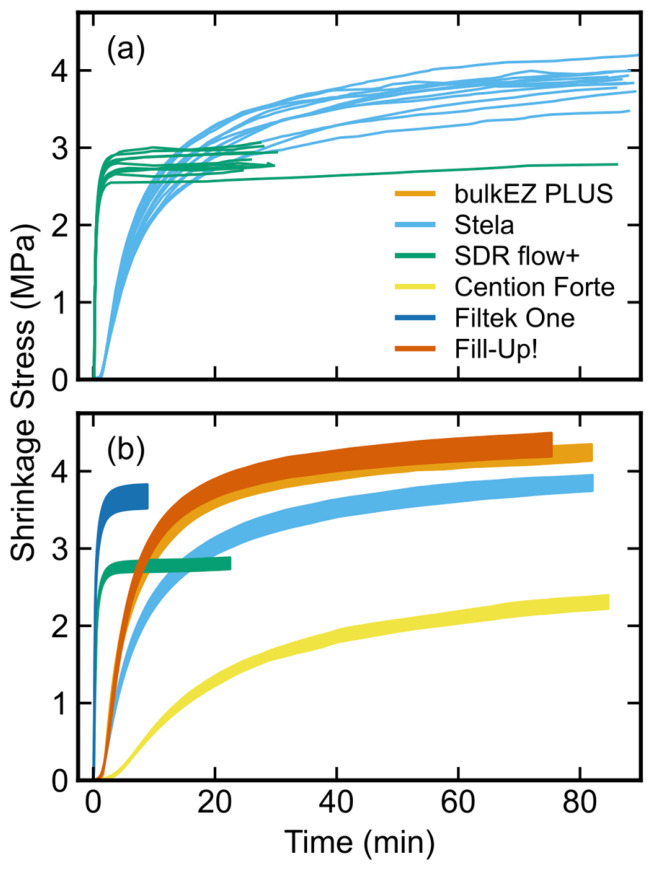
Axial shrinkage stress of the six RBCs. (**a**) Shrinkage stress over time for Stela Capsule (self-cured) and SDR flow+ (light-cured). The SDR flow+ example recorded for 4000 s showed little change from 1400 s to 4000 s. (**b**) Mean shrinkage stress over time for four self-cured (n = 12 repeats) and two light-cured RBCs (n = 11 repeats); the line thickness represents ± 2 standard errors (SE), so the total thickness represents 4 SE.

The SS development profiles differed markedly between the light-cured and self-cured RBCs (Figure 3). The two light-cured RBCs (SDR flow+ and Filtek One) developed a rapid increase in stress immediately after light activation and reached an early plateau within the first 20 s. In contrast, the four self-cured materials showed a more gradual, sustained increase in stress over time, with values continuing to rise throughout the 4000 s observation period. Among the self-cured RBCs, Bulk EZ Plus and Fill-Up! generated the highest final stress values, followed by Stela, while Cention Forte exhibited substantially lower stress throughout the entire observation period. The individual traces for Stela showed a consistent polymerization behavior across the repeats, with limited variability (Figure 3a).

### 3.3. Degree of Conversion (DC) and Vickers Hardness (HV)

**Figure 4:**
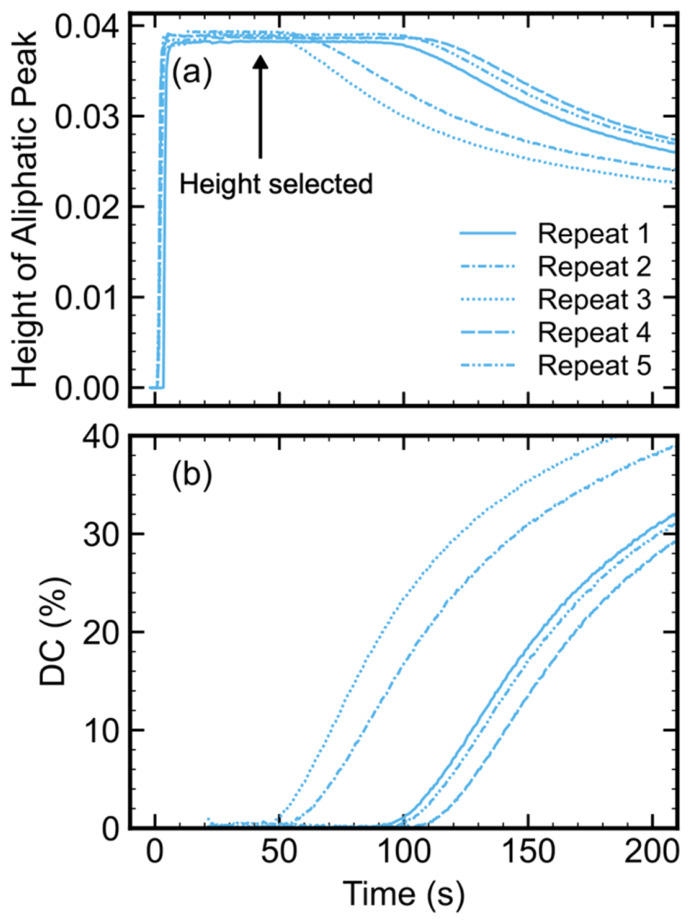
Stela aliphatic peak height and DC over time. (**a**) The aliphatic peak height remained stable for approximately 100 s after placement, then decreased as polymerization began. The vertical arrow marks the pre-polymerization peak height, defined as H(t = 0), used for DC calculations. (**b**) DC as a function of time for five repeats.

Figure 4 shows the aliphatic peak height and the DC over time for the self-cured Stela RBC. After placement on the ATR diamond prism, the aliphatic peak height remained stable for 40 to 100 s before it began to decrease, indicating the onset of polymerization. The vertical arrow in Figure 4a marks the pre-polymerization peak height used as H(t = 0) in Equation (2). For each repeat, the DC curve in Figure 4b was shifted so that t = 0 corresponded to the start of polymerization. The peak height traces are shown as originally collected, whereas the variation in the aligned DC curves reflects intrinsic differences among the Stela specimens. A similar pattern was observed for the other RBCs tested.

**Figure 5:**
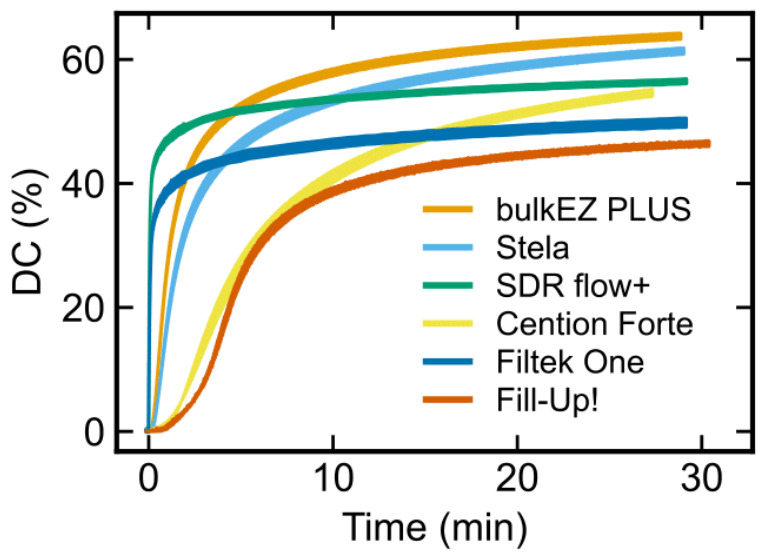
Real-time DC for the six RBCs. The DC was monitored continuously for 1800 s from the onset of polymerization using ATR-FTIR spectroscopy. Note that the DC data of self-cured RBCs have been shifted in time. Curves shown are the average DC(t) values, and the line thickness represents ± 2 standard errors (SE), so the total thickness represents 4 SE.

Figure 5 shows real-time DC for the six RBCs and reveals distinct polymerization profiles. Bulk EZ Plus and Stela showed rapid initial conversion and reached the highest final DC values within the first minutes, whereas SDR flow+ rose rapidly but then plateaued at a slightly lower DC. Cention Forte and Fill-Up! had slower, more progressive conversion profiles, and Filtek One showed an intermediate polymerization profile followed by a lower plateau.

The DC parameters differed significantly among the RBCs for all outcomes (*p* < 0.001, Table 3). Overall, the light-cured RBCs were characterized by rapid polymerization kinetics, while self-cured RBCs had slower conversion profiles. At 1600 s, Bulk EZ Plus produced the highest DC (63.29%), followed by Stela (60.67%), whereas Fill-Up! produced the lowest value (45.76%). The maximum DC rate was substantially higher for the light-cured materials (SDR flow+ and Filtek One) than for the self-cured materials, with SDR flow+ generating the highest rate. The self-cured materials reached their maximum DC rate at significantly longer times, particularly for Cention Forte and Fill-Up!. In contrast, the light-cured materials reached their peak rates almost immediately after exposure to the curing light. The DC at the time of maximum rate was highest for Fill-Up! and Cention Forte, intermediate for Bulk EZ Plus and Stela, and lowest for the light-cured materials.

**Figure 6:**
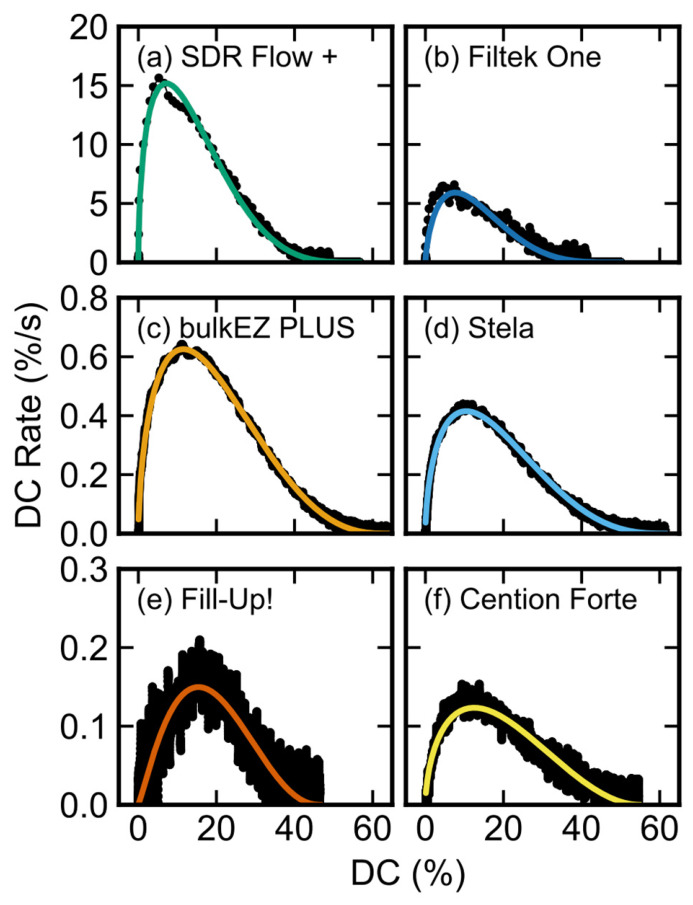
DC rate as a function of DC for the six RBCs, along with the autocatalytic fit. Note that different y-axis scales were used to accommodate the varying rates. The results shown are the interpolated, averaged, and smoothed data from the five repeated measurements.

Figure 6 shows the DC rate as a function of DC for the six RBCs, together with the autocatalytic model used to fit the data. The interpolated, averaged, and smoothed data are shown. The two light-cured RBCs had the largest peak polymerization rates: 14.91 ± 0.42%/s for SDR flow+ and 6.13 ± 0.54%/s for Filtek One. The lowest peak rate was 0.13 ± 0.03%/s for Cention Forte. The DC at which the maximum rate occurred, DC(t_0_), was lower for the light-cured RBCs (8.60 ± 0.35% for SDR flow+ and 7.24 ± 0.29% for Filtek One) than for the self-cured RBCs, which ranged from 10.46 ± 0.38% for Stela to 15.40 ± 1.03% for Fill-Up!. Figure 6 illustrates how the autocatalytic model described the data for both self-cured and light-cured RBCs.

Table 4 reports the HV data of the six resin-based composites measured on top and bottom surfaces. The hardness differed significantly among materials for all outcomes (*p* < 0.001). On the top surface, Stela (84.56 ± 0.94 HV) and Filtek One (79.27 ± 2.25 HV) produced the highest hardness values, followed by Cention Forte (73.58 ± 2.17 HV) and Fill-Up! (57.43 ± 0.57 HV), while SDR flow+ (48.66 ± 0.99 HV) and Bulk EZ Plus (43.00 ± 2.74 HV) showed the lowest values. A similar trend was observed on the bottom surface, with Stela (80.75 ± 1.85 HV) and Filtek One (68.14 ± 2.18 HV) maintaining higher hardness, whereas SDR flow+ (39.12 ± 0.96 HV) and Bulk EZ Plus (43.16 ± 0.95 HV) remained the lowest.

The top/bottom hardness difference also varied significantly among materials (*p* < 0.001). Cention Forte showed the greatest reduction (16.32 ± 3.65 HV), followed by Filtek One (11.13 ± 3.11 HV) and SDR flow+ (9.54 ± 1.06 HV), while Bulk EZ Plus showed no meaningful difference between surfaces (−0.16 ± 3.07 HV). Paired comparisons confirmed significantly higher top hardness than bottom hardness for Stela (*p* = 0.020) and Cention Forte (*p* = 0.003), for Fill-Up! (*p* < 0.001) and Filtek One (*p* = 0.008), whereas no significant differences were observed for Bulk EZ Plus (*p* = 1.000) and SDR flow+ (*p* = 0.354).

**Figure 7:**
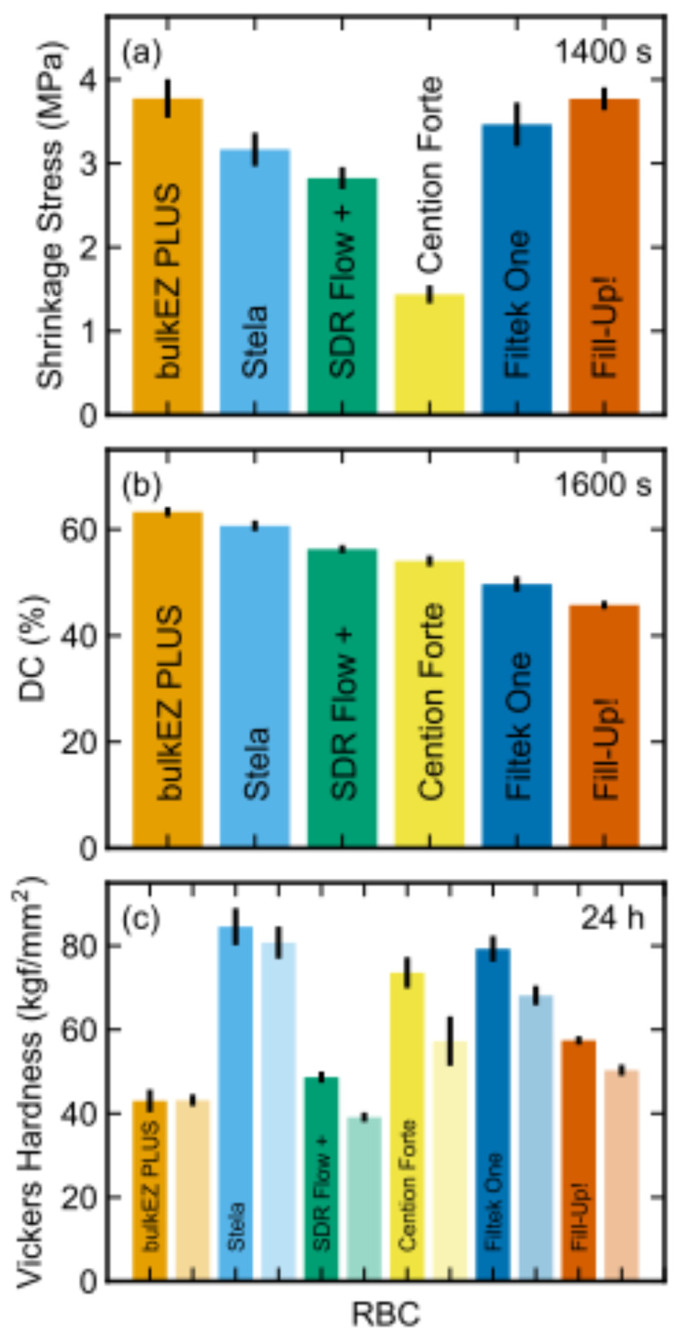
(**a**) Shrinkage stress after 1400 s, (**b**) DC after 1600 s, and (**c**) top and bottom HV of the six RBCs, with materials shown in the same color across the panels. Left and right bars represent the top and bottom surfaces of the same RBC. Error bars indicate ±1 standard error. The statistical analysis for these data is provided in Table 2, Table 3 and Table 4.

Figure 7 summarizes shrinkage stress, DC, and HV. At 1400 s, Bulk EZ Plus, Filtek One, and Fill-Up! produced the highest stress, Stela and SDR flow+ were intermediate, and Cention Forte was the lowest. At 1600 s, Bulk EZ Plus reached the highest DC, followed by Stela and SDR flow+, whereas Fill-Up! had the lowest DC. For HV after 24 h, Stela had the highest hardness among the self-cured RBCs, followed by Cention Forte, Fill-Up!, and Bulk EZ Plus; among the light-cured RBCs, Filtek One was harder than SDR flow+.

**Figure 8:**
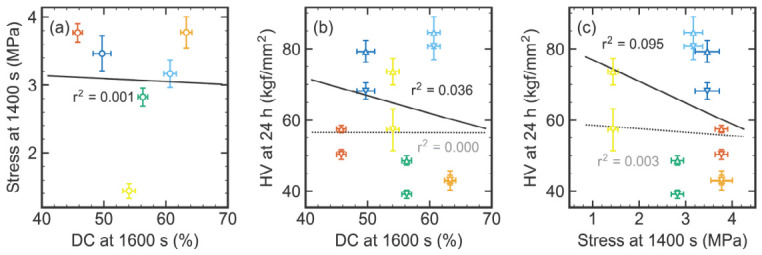
Regression lines and r^2^ values for: (**a**) shrinkage stress at 1400 s and DC at 1600 s, (**b**) Vickers hardness at the top (solid line) and bottom surfaces (dashed line) at 24 h and DC at 1600 s, or (**c**) Vickers hardness at the top (solid line) and bottom surfaces (dashed line) at 24 h and shrinkage stress at 1400 s. The colors used are the same as in Figure 5, Figure 6 and Figure 7. Error bars indicate two standard errors.

Regression analysis of the raw values showed no meaningful relationship among the measured outcomes (Figure 8). Shrinkage stress at 1400 s was not correlated with DC at 1600 s (r^2^ = 0.001). Similarly, HV at 24 h was not correlated with DC at 1600 s (r^2^ = 0.036 for the top surface and r^2^ = 0.000 for the bottom surface) or with shrinkage stress at 1400 s (r^2^ = 0.095 for the top surface and r^2^ = 0.003 for the bottom surface).

## 4. Discussion

This study compared the shrinkage stress of four RBCs used in their self-cured mode and two light-cured controls, using standardized 6 mm diameter by 2 mm thick specimens. The real-time development of SS and DC, together with 24 h HV, differed significantly among the materials; therefore, all three null hypotheses were rejected. Within the limitations of this *in vitro* study, it was confirmed that the polymerization behavior of these bulk-fill RBCs was strongly material-dependent [6,9,12,15,16,17,18,19,20,21,35,51] rather than determined simply by whether the material was self-cured or light-cured.

The SS results showed that self-cured and dual-cured materials used in self-curing mode should not be treated as a single category. Cention Forte developed the lowest shrinkage stress and the slowest stress development rate, whereas Bulk EZ Plus and Fill-Up! generated the highest shrinkage stresses. Stela showed a favorable balance of high DC, high HV, relatively uniform top-to-bottom hardness, and moderate SS (Table 2, Table 3 and Table 4), consistent with previous findings [12]. The filler-volume data in Table 1 do not alone explain these differences. Instead, the stress response is more likely related to the combined effects of resin–matrix chemistry, filler morphology, viscosity, initiator system, and the rate at which the polymer network formed [12]. The slower stress-development and polymerization rates observed for Cention Forte may have allowed more stress relaxation during network formation, whereas the faster stress development rates of Bulk EZ Plus and Fill-Up! suggest earlier network formation and fewer opportunities for relaxation.

The two light-cured RBCs, Filtek One and SDR flow+, developed shrinkage stress rapidly after irradiation and reached an early plateau (Figure 3). This is consistent with the rapid reaction rate and network formation typical of photopolymerized RBCs, which reduces the time available for flow and stress relaxation [1]. Even small increases in the temperature of the RBC can accelerate polymerization and increase monomer mobility before vitrification [52]. Transient thermal effects may also have influenced the subsequent plateau because early polymerization shrinkage can be partially masked by thermal expansion caused by the exothermic reaction and the temperature rise produced by the LCU [22]. In this study, the self-curing materials received no external energy input from LCUs [53], and they exhibited a more gradual stress-development profile. Their longer monitoring period (4000 s) reflected slower polymerization kinetics and extended pre-gel phases [12], consistent with reports that self-curing RBCs can exhibit lower shrinkage force because of differences in polymerization kinetics [22,39,54].

SDR flow+ produced lower SS than Filtek One. This may be related to its UDMA-based resin chemistry, because UDMA has relatively low viscosity, greater molecular flexibility, and contains an amino (-NH-) group that can facilitate chain-transfer reactions [20]. Delayed resin immobilization may allow conversion to continue for longer before network mobility becomes restricted [12,55]. These findings support the view that SS is influenced not only by viscosity and curing mode [54], but also by monomer composition [6]. Bulk EZ Plus and Stela also contain UDMA (Table 1) and had relatively high DC values, but their stress and hardness values differed, again indicating that composition and network development affect the measured outcomes in different ways.

Although the primary focus of this study was to report the shrinkage stress, the DC and Vickers hardness of the RBCs were also reported because an inadequately polymerized RBC will produce less shrinkage stress. However, it must be recognized that the DC is not an absolute measure of polymer formation or complete curing, and because RBCs have different compositions, DC values should not be compared across RBCs. However, changes in DC within the RBC as it polymerizes can be used to monitor the polymerization reaction, because the DC value is a relative percentage reduction in the absorbance of methacrylate double bonds compared to the uncured RBC. Therefore, when a stable aromatic band is present, the mid-IR DC can be calculated from the reduction in the methacrylate C=C band near 1635–1638 cm^−1^ and normalized to the aromatic band near 1607–1609 cm^−1^. For materials without an identifiable aromatic reference peak, conversion can be calculated from the change in the methacrylate C=C peak alone [45,46]. In this study, the light-cured RBCs converted rapidly due to light activation and thus did not have to be measured for 4000 s. However, they did not have the highest DC after 1600 s. Bulk EZ Plus and Stela achieved the highest DC values, while Cention Forte and Fill-Up! showed slower polymerization and lower DC (Table 3 and Figure 5). Stela also contains glycerol dimethacrylate (GDMA), which may contribute to conversion, cross-linking, and mechanical properties [35]. Figure 8 illustrates the lack of correlation among these outcomes: DC at 1600 s did not correlate with shrinkage stress at 1400 s (r^2^ = 0.001) and explained little or none of the variation in HV at 24 h (r^2^ = 0.036 for the top surface and r^2^ = 0.000 for the bottom surface).

The HV results also differed among materials and between top and bottom surfaces, leading to rejection of the third hypothesis. Hardness was affected by both monomer conversion and filler characteristics [22]. The largest top–bottom difference was observed for Cention Forte, while the effects of light attenuation may have contributed to the bottom surface reduction observed for Filtek One [19,20,25,26,27]. Temperature gradients during polymerization may also have contributed to surface differences because the bottom surface was positioned on the ATR plate at 23 °C. For the light-cured RBCs, the top surface also received the full thermal input from the curing light. Stela had the highest overall hardness, exceeding even that of the light-cured Filtek One (Table 4). The regression analysis also showed that HV at 24 h did not correlate with shrinkage stress at 1400 s (r^2^ = 0.095 for the top surface and r^2^ = 0.003 for the bottom surface; Figure 8). Thus, a high hardness value did not necessarily indicate a higher DC across these materials [56]. These data indicate that DC, HV, and SS values measured in this study were not related across the six RBCs tested (Figure 8); however, the DC and HV data did show that the RBCs had reached their own respective maximum DC and HV values.

The clinical relevance of these findings should be interpreted cautiously. The totally self-cured options evaluated in this study are required when light access is limited, such as in deep proximal boxes, post-cementation, or beneath indirect restorations. Cention Forte had the lowest SS and the slowest polymerization kinetics, which could be advantageous in high C-factor situations where stress relaxation is relevant to marginal integrity [1,13,14]. However, light activation of Cention Forte is recommended to improve DC [12]. Bulk EZ Plus achieved the highest DC and showed uniform top-to-bottom hardness, but its higher SS could increase interfacial stress in highly constrained cavities [57]. Fill-Up! showed high SS combined with lower final DC, suggesting that optional light activation may be important for maximizing polymerization [12]. Two clinical trials have already reported favorable results for Stela [40,41], and Stela had the most favorable overall balance among the evaluated factors in the present study. However, no measurements of cavity adaptation, marginal leakage, bond strength, or clinical outcomes were made in this study. Therefore, the results of the present study apply only to the test conditions, the RBCs used in this study, the limited number of repetitions (n = 5) for the DC and HV, the specimen geometry, the curing protocols, and the time points examined. Future studies should evaluate volumetric shrinkage, modulus development, longer post-polymerization behavior, different specimen thicknesses, and clinically relevant cavity configurations [57].

## 5. Conclusions

Within the limitations of this *in vitro* study:

1. The axial shrinkage stress, degree of conversion, and Vickers hardness differed among the six resin-based composites.
2. Cention Forte generated the lowest shrinkage stress, whereas Bulk EZ Plus and Fill-Up! generated the highest stress values; therefore, the self-curing mode did not uniformly reduce shrinkage stress for all the products tested.
3. The degree of conversion, hardness, and shrinkage stress were unrelated and not predictors of material behavior.
4. These findings apply only to the resin-based composites, the specimen geometry, the curing conditions, and the time points tested. Future work should include volumetric shrinkage, longer post-polymerization measurements, additional specimen thicknesses, and clinically relevant thermal conditions.

## Figures and Tables

**Table 1:** Composition of the RBCs evaluated as provided by the manufacturers.

Material	Manufacturer	Resin Matrix and Additive Composition	Filler Composition	Filler Content % (vol)/(wt)	LOT Number
Stela Capsule	SDI, Bayswater, Australia	Methacrylates (23 wt%): UDMA, Glycerol dimethacrylate, 10-MDP Initiators, stabilizers, pigments (<1 wt%)	Fluoro-alumino-silicate glass, Ytterbium trifluoride, Silicon dioxide (hydrophobic fumed silica), Calcium aluminate	55/76	1241402
Cention Forte	Ivoclar, Schaan, Liechtenstein	Copolymer, UDMA, aromatic aliphatic UDMA, DCP, PEG-DMA	Ca-fluorosilicate glass, Ba-Al silicate glass, Ca-Ba-Al fluorosilicate glass, ytterbium trifluoride. Particle size between 0.1–7 µm	58–59/NA	ZL12C5
Bulk EZ Plus	Zest Dental Solutions, Carlsbad, CA, USA	Ethoxylated bisphenol A dimethacrylate, Triethylene glycol dimethacrylate, Bisphenol A glycidyl methacrylate, Diurethane dimethacrylate, Initiator	zirconia-silica filler, Radiopaque filler	60–70/NA	L33KQ
Fill-Up!	Coltene, Altstätten, Switzerland	Methacrylates	Dental glass, amorphous silica, zinc oxide	49/65	N21189
SDR flow+	Dentsply Sirona, Milford, DE, USA	Modified UDMA resin, EBPADMA, TEGDMA, Camphorquinone, Photoinitiator, photoaccelerator, BHT, UV stabilizer	Barium-alumino-fluoro-borosilicate glass, strontium alumino-fluoro-silicate glass, Titanium dioxide. Particles of inorganic filler range from 20 nm to 10 µm	47.4/70.5	00117209 and 00120987
Filtek One	Solventum (formerly 3M), St. Paul, MN, USA	AFM (stress-relieving monomer), AUDMA, UDMA, 1,12-dodecane DMA	20 nm silica, 4–11 nm zirconia, zirconia/silica cluster filler, ytterbium trifluoride (100 nm)	58.5/76.5	10732823 and 11541817

Abbreviations: UDMA, urethane dimethacrylate; 10-MDP, 10-methacryloyloxydecyl dihydrogen phosphate; DCP, tricyclodecane-dimethanol dimethacrylate; PEG-DMA, Poly(ethylene glycol) dimethacrylate; EBPADMA, ethoxylated Bisphenol A dimethacrylate; TEGDMA, Triethylene Glycol dimethacrylate; BHT, Butylated hydroxytoluene; AFM, addition fragmentation monomers; AUDMA, aromatic urethane dimethacrylate; NA, not available.

**Table 2:** Mean Shrinkage Stress (±Standard Error) Values of the six RBCs.

RBC	Stress at 1400 s (MPa)	Group	Stress at 4000 s (MPa)	Group	Max Stress Rate (MPa/s)	Group	Time of Max Stress Rate (s)	Group	Stress at Max Rate (MPa)	Group
Stela	3.17 (0.20)	a	3.77 (0.19)	a	0.0074 (0.0009)	a	147.41 (8.70)	ab	0.49 (0.06)	a
Cention Forte	1.44 (0.11)	b	2.18 (0.15)	b	0.0016 (0.0003)	b	432.36 (35.67)	c	0.39 (0.04)	b
Bulk EZ Plus	3.77 (0.23)	c	4.19 (0.19)	c	0.0130 (0.0022)	c	132.09 (3.32)	a	0.48 (0.04)	a
Fill-Up!	3.77 (0.14)	c	4.19 (0.20)	c	0.0113 (0.0021)	c	172.84 (16.57)	bc	0.81 (0.09)	c
SDR flow+	2.82 (0.13)	d	Not Measured		Not Measured		Not Measured		Not Measured	
Filtek One	3.46 (0.26)	e	Not Measured		Not Measured		Not Measured		Not Measured	

Each value represents the mean (±standard error). Stress values at 1400 s and 4000 s (MPa), maximum stress rate (MPa/s), time of maximum stress rate (s), and stress at the time of maximum rate (MPa). For SDR flow+ and Filtek One, stress at 4000 s and stress-rate-based metrics were not measured because these light-cured materials reached a rapid plateau; therefore, only the 1400 s stress values are reported. At 1400 s, SDR flow+ and Filtek One were based on 11 repeats; all others were based on 12 repeats. CLD group letters are from post hoc pairwise comparisons (see Supplementary Materials); materials sharing a letter are not significantly different (*α* = 0.05).

**Table 3:** Mean Degree of Conversion (DC) ± (Standard Error) Values of the six RBCs.

RBC	DC at 1600 s (%)	Group	Max DC Rate (%/s)	Group	Time of Max DC Rate (s)	Group	DC at Max Rate (%)	Group
Stela	60.67 (1.00)	ab	0.42 (0.05)	ab	25.41 (3.09)	ab	10.46 (0.38)	abc
Cention Forte	54.06 (1.01)	abc	0.13 (0.03)	a	101.49 (30.42)	a	12.34 (1.63)	ab
Bulk EZ Plus	63.29 (0.91)	a	0.63 (0.04)	ab	18.56 (1.04)	ab	11.59 (0.36)	ab
Fill-Up!	45.76 (0.76)	c	0.15 (0.02)	a	101.67 (9.24)	a	15.40 (1.03)	a
SDR flow+	56.29 (0.78)	abc	14.91 (0.42)	b	0.58 (0.02)	b	8.60 (0.35)	bc
Filtek One	49.71 (1.44)	bc	6.13 (0.54)	b	1.18 (0.07)	b	7.24 (0.29)	c

Each value represents the mean (±standard error) across five specimens per material. DC at 1600 s (%), maximum DC rate (%/s), time of maximum DC rate (s), and DC at the time of maximum rate (%) were computed from specimen-level DC time curves and summarized across specimens. CLD group letters are from post hoc pairwise comparisons (see Supplementary Materials); materials sharing a letter are not significantly different (*α* = 0.05).

**Table 4:** Mean Vickers Hardness (HV) ± (Standard Error) of the six RBCs.

RBC	Top Surface (HV)	Group	Bottom Surface (HV)	Group	Top − Bottom (HV)	Group
Stela	84.56 (0.94)	a	80.75 (1.85)	a	3.80 (1.35)	ab
Cention Forte	73.58 (2.17)	abc	57.26 (2.96)	abc	16.32 (3.65)	c
Bulk EZ Plus	43.00 (2.74)	b	43.16 (0.95)	bc	−0.16 (3.07)	a
Fill-Up!	57.43 (0.57)	abc	50.33 (0.68)	abc	7.10 (1.04)	abc
SDR flow+	48.66 (0.99)	bc	39.12 (0.96)	b	9.54 (1.06)	bc
Filtek One	79.27 (2.25)	ac	68.14 (2.18)	ac	11.13 (3.11)	bc

Each value represents the mean (±Standard Error) across five specimens of each RBC. For each sample, the top and bottom values are the means of five Vickers indentations per surface; top-surface hardness (HV), bottom-surface hardness (HV), and the top−bottom difference (HV) were each computed at the sample level and summarized across samples. CLD group letters are from post hoc pairwise comparisons (see Supplementary Materials); RBCs sharing the same letter are not significantly different (*α* = 0.05).

## Data Availability

The raw data supporting the conclusions of this article are available from the authors on request.

## Supplementary Materials

The following supporting information can be downloaded at: https://www.mdpi.com/article/10.3390/ma19122623/s1, Table S1. Shrinkage Stress Parameters of Resin-Based Composites; Table S2. Omnibus Tests and Post-Hoc Comparisons for Shrinkage Stress Parameters; Table S3. Vickers Hardness (HV) of Resin-Based Composites; Table S4. Omnibus Tests and Post-Hoc Comparisons for Vickers Hardness; Table S5. Degree of Conversion (DC) Parameters of Resin-Based Composites; Table S6. Omnibus Tests and Post-Hoc Comparisons for DC Parameters.
