# Supplementary material for: Shrinkage Stress, Polymerization Kinetics, and Hardness of Light and Self-Cured Bulk-Fill Resin-Based Composites
Source: Materials (Basel). 2026 Jun 18;19(12):2623. doi: 10.3390/ma19122623 (PMC13303416; doi:10.3390/ma19122623)
Supplement: Supplementary file 1 [file materials-19-02623-s001.zip › materials-4332554-supplementary.pdf]

Shrinkage Stress

Table S1. Shrinkage Stress Parameters of Resin-Based Composites

Each value represents the mean (SD). Stress values at 1,400 s and 4,000 s (MPa), the per-specimen stress change from 1,400 s to 4,000 s (Δ, MPa), maximum stress rate (MPa/s), time of maximum stress rate (s), and stress at the time of maximum rate (MPa). For SDR flow+ and Filtek One, stress-rate-based metrics and stress at 4,000 s and the 4,000 s − 1,400 s change were not applicable (—). At 1,400 s, SDR flow+ and Filtek One are based on 11 repeats; all others on 12 repeats. CLD group letters are from post-hoc pairwise comparisons (see Table S2); materials sharing a letter are not significantly different ($\alpha = 0.05$).

RBC	Stress at 1,400 s (MPa)	Group	Stress at 4,000 s (MPa)	Group	Change 4,000−1,400 s (MPa)	Group	Max Stress Rate (MPa/s)	Group	Time of Max Stress Rate (s)	Group	Stress at Max Rate (MPa)	Group
Stela	3.17 (0.20)	a	3.77 (0.19)	a	0.61 (0.12)	a	0.0074 (0.0009)	a	147.41 (8.70)	ab	0.49 (0.06)	a
Cention Forte	1.44 (0.11)	b	2.18 (0.15)	b	0.75 (0.11)	b	0.0016 (0.0003)	b	432.36 (35.67)	c	0.39 (0.04)	b
BulkEZ PLUS	3.77 (0.23)	c	4.19 (0.19)	c	0.41 (0.10)	c	0.0130 (0.0022)	c	132.09 (3.32)	a	0.48 (0.04)	a
Fill-Up!	3.77 (0.14)	c	4.19 (0.20)	c	0.42 (0.13)	c	0.0113 (0.0021)	c	172.84 (16.57)	bc	0.81 (0.09)	c
SDR flow+	2.82 (0.13)	d	—		—		—		—		—	
Filtek One	3.46 (0.26)	e	—		—		—		—		—	

Table S2. Omnibus Tests and Post-Hoc Comparisons for Shrinkage Stress Parameters

Measure	Groups (k)	Test chosen	Justification	Omnibus statistic	p-value	Effect size
Stress at 1,400 s	6 (all RBCs)	One-way ANOVA	Normality met (all SW $p \geq 0.05$, residuals $p = 0.999$); equal variances (Levene's $p = 0.101$)	$F(5, 64) = 267.785$	< 0.001	$\eta^2 = 0.954$
Stress at 4,000 s	4 (self-cured)	Kruskal-Wallis	Fill-Up! failed SW ($p < 0.05$); variances equal (Levene's $p = 0.871$)	$H(3) = 37.585$	< 0.001	$\eta^2 = 0.786$
1,400 s vs. 4,000 s (within-RBC, paired)	4 (self-cured)	Paired t-test (all 4)	SW on paired Δ: all p ≥ 0.100; Bonf. correction across 4 RBCs	$t(11) = 11.32–22.49$	< 0.001 (all; Bonf. adj.)	$d_z = 3.27–6.49$
Stress Change 4,000−1,400 s	4 (self-cured)	One-way ANOVA	All SW p ≥ 0.100; residuals W = 0.987, p = 0.850; Levene's p = 0.931	$F(3, 44) = 22.911$	< 0.001	$\eta^2 = 0.610$
Max Stress Rate	4 (self-cured)	Welch's ANOVA	Per-group normality met (all SW $p \geq 0.05$); residual non-normality ($W = 0.941$, $p = 0.017$) attributed to unequal variances	$F(3.0, 19.7) = 281.630$	< 0.001	$\eta^2 = 0.889$

Measure	Groups (k)	Test chosen	Justification	Omnibus statistic	p-value	Effect size
			(Levene's $p < 0.001$)			
Time of Max Stress Rate	4 (self-cured)	Kruskal-Wallis	CF and BulkEZ PLUS failed SW; unequal variances (Levene's $p < 0.001$)	$H(3) = 42.450$	< 0.001	$\eta^2 = 0.897$
Stress at Max Rate	4 (self-cured)	Welch's ANOVA	Normality met (all SW $p \geq 0.05$, residuals $p = 0.992$); unequal variances (Levene's $p = 0.026$)	$F(3.0, 23.7) = 73.400$	< 0.001	$\eta^2 = 0.884$

Vickers Hardness

Table S3. Vickers Hardness (HV) of Resin-Based Composites

Each value represents the mean (SD) across five samples per material. For each sample, top and bottom values are the mean of five Vickers indentations per surface; top surface hardness (HV), bottom surface hardness (HV), and the top−bottom difference (HV) were each computed at the sample level and summarized across samples. CLD group letters are from post-hoc pairwise comparisons (see Table S4); materials sharing a letter are not significantly different (α = 0.05).

RBC	Top Surface (HV)	Group	Bottom Surface (HV)	Group	Top − Bottom (HV)	Group
Stela	84.56 (0.94)	a	80.75 (1.85)	a	3.80 (1.35)	ab
Cention Forte	73.58 (2.17)	abc	57.26 (2.96)	abc	16.32 (3.65)	c
BulkEZ PLUS	43.00 (2.74)	b	43.16 (0.95)	bc	−0.16 (3.07)	a
Fill-Up!	57.43 (0.57)	abc	50.33 (0.68)	abc	7.10 (1.04)	abc
SDR flow+	48.66 (0.99)	bc	39.12 (0.96)	b	9.54 (1.06)	bc
Filtek One	79.27 (2.25)	ac	68.14 (2.18)	ac	11.13 (3.11)	bc

Table S4. Omnibus Tests and Post-Hoc Comparisons for Vickers Hardness

Measure	Test chosen	Justification	Omnibus statistic	*p*-value	Effect size
Top-surface HV (k = 6)	Kruskal-Wallis	Non-normality in residuals (p < 0.001)	$H(5) = 27.978$	< 0.001	$\eta^2 = 0.957$
Bottom-surface HV (k = 6)	Kruskal-Wallis	Non-normality in residuals (p < 0.001)	$H(5) = 28.226$	< 0.001	$\eta^2 = 0.968$
Top vs. Bottom (paired within RBC, n = 5)	Paired *t*-test / Wilcoxon	Selected per RBC based on SW normality of differences	See below		
Top−Bottom Diff (k = 6)	Kruskal-Wallis	Non-normality (p < 0.001)	$H(5) = 26.125$	< 0.001	$\eta^2 = 0.880$

Paired top vs. bottom results (Bonferroni-corrected across 6 RBCs):

RBC	Primary test	Test statistic	Unadj. *p*	Bonf. adj. *p*	Cohen's d_z	Significant?
Stela	Paired *t*	$t(4) = 6.281$	0.003	0.020	2.81	Yes
Cention Forte	Paired *t*	$t(4) = 9.986$	0.001	0.003	4.47	Yes
BulkEZ PLUS	Paired *t*	$t(4) = -0.114$	0.915	1.000	−0.05	No
Fill-Up!	Paired *t*	$t(4) = 15.267$	< 0.001	< 0.001	6.83	Yes
SDR flow+	Wilcoxon	$W = 15.000$	0.059	0.354	8.98	No
Filtek One	Paired *t*	$t(4) = 8.000$	0.001	0.008	3.58	Yes

Degree of Conversion

Table S5. Degree of Conversion (DC) Parameters of Resin-Based Composites

Each value represents the mean (SD) across five specimens per material. DC at 1,800 s (%), maximum DC rate (%/s), time of maximum DC rate (s), and DC at the time of maximum rate (%) were computed from specimen-level DC time curves and summarized across specimens. CLD group letters are from post-hoc pairwise comparisons (see Table S6); materials sharing a letter are not significantly different (α = 0.05).

RBC	DC at 1,800 s (%)	Group	Max DC Rate (%/s)	Group	Time of Max DC Rate (s)	Group	DC at Max Rate (%)	Group
Stela	60.67 (1.00)	ab	0.42 (0.05)	ab	25.41 (3.09)	ab	10.46 (0.38)	abc
Cention Forte	54.06 (1.01)	abc	0.13 (0.03)	a	101.49 (30.42)	a	12.34 (1.63)	ab
BulkEZ PLUS	63.29 (0.91)	a	0.63 (0.04)	ab	18.56 (1.04)	ab	11.59 (0.36)	ab
Fill-Up!	45.76 (0.76)	c	0.15 (0.02)	a	101.67 (9.24)	a	15.40 (1.03)	a
SDR flow+	56.29 (0.78)	abc	14.91 (0.42)	b	0.58 (0.02)	b	8.60 (0.35)	bc
Filtek One	49.71 (1.44)	bc	6.13 (0.54)	b	1.18 (0.07)	b	7.24 (0.29)	c

Table S6. Omnibus Tests and Post-Hoc Comparisons for DC Parameters

Measure	Test chosen	Justification	Omnibus statistic	*p*-value	Effect size
DC at 1,800 s (*k* = 6)	Kruskal-Wallis	Non-normality (≥1 group failed SW at n = 5); equal variances (Levene's $p = 0.981$)	$H(5) = 28.226$	< 0.001	$\eta^2 = 0.968$
Max DC Rate (*k* = 6)	Kruskal-Wallis	Non-normality (residuals $p < 0.001$)	$H(5) = 27.524$	< 0.001	$\eta^2 = 0.939$
Time of Max DC Rate (*k* = 6)	Kruskal-Wallis	Non-normality (residuals $p < 0.001$)	$H(5) = 27.431$	< 0.001	$\eta^2 = 0.935$
DC at Max Rate (*k* = 6)	Kruskal-Wallis	Non-normality (residuals $p < 0.001$)	$H(5) = 27.142$	< 0.001	$\eta^2 = 0.923$
